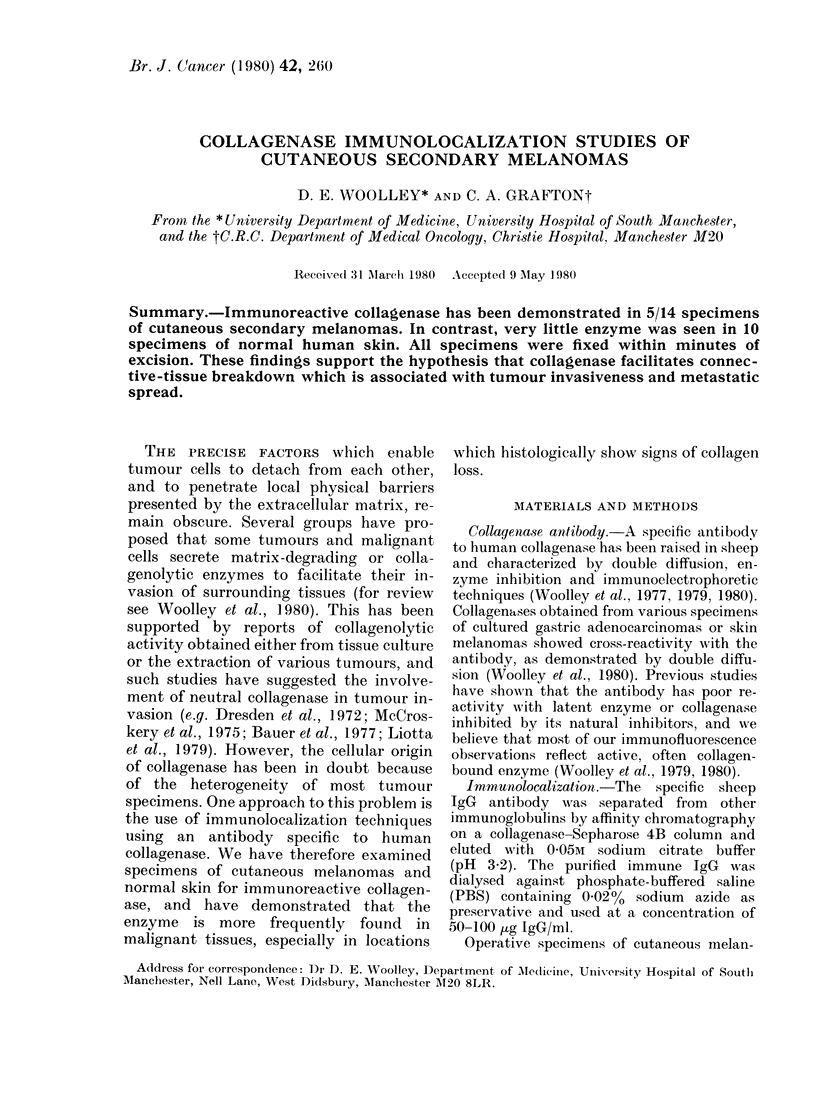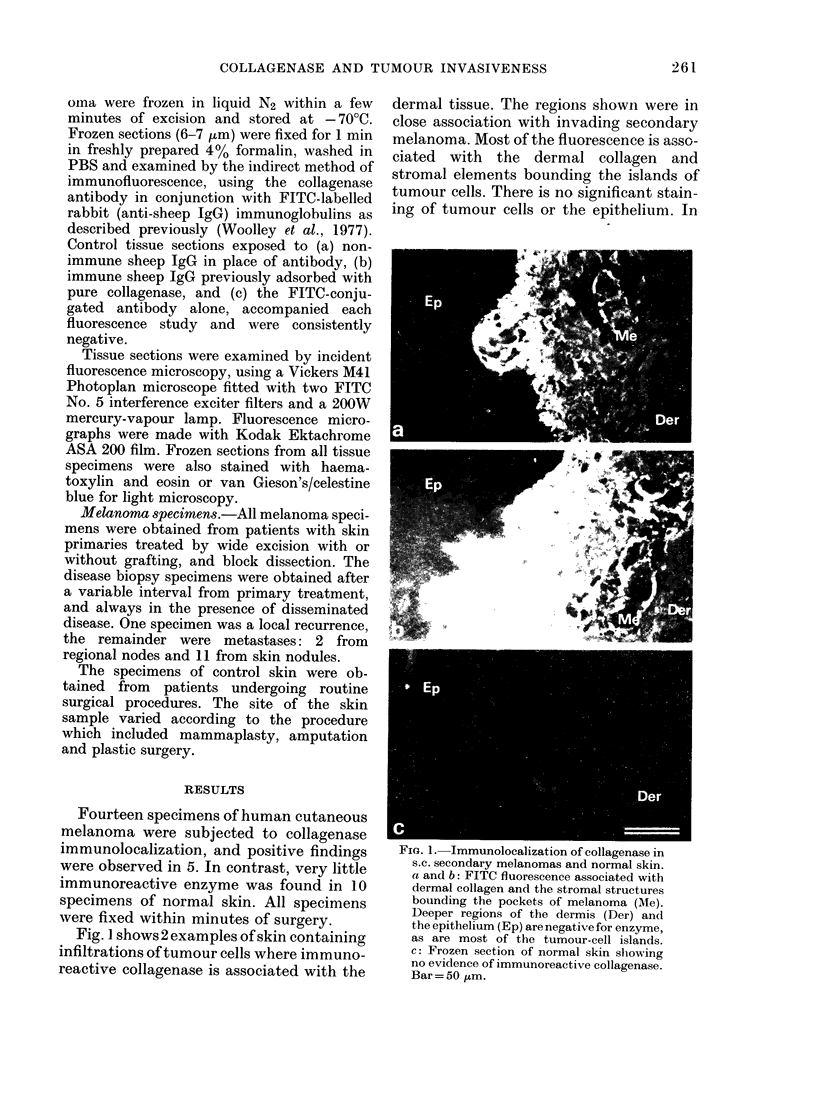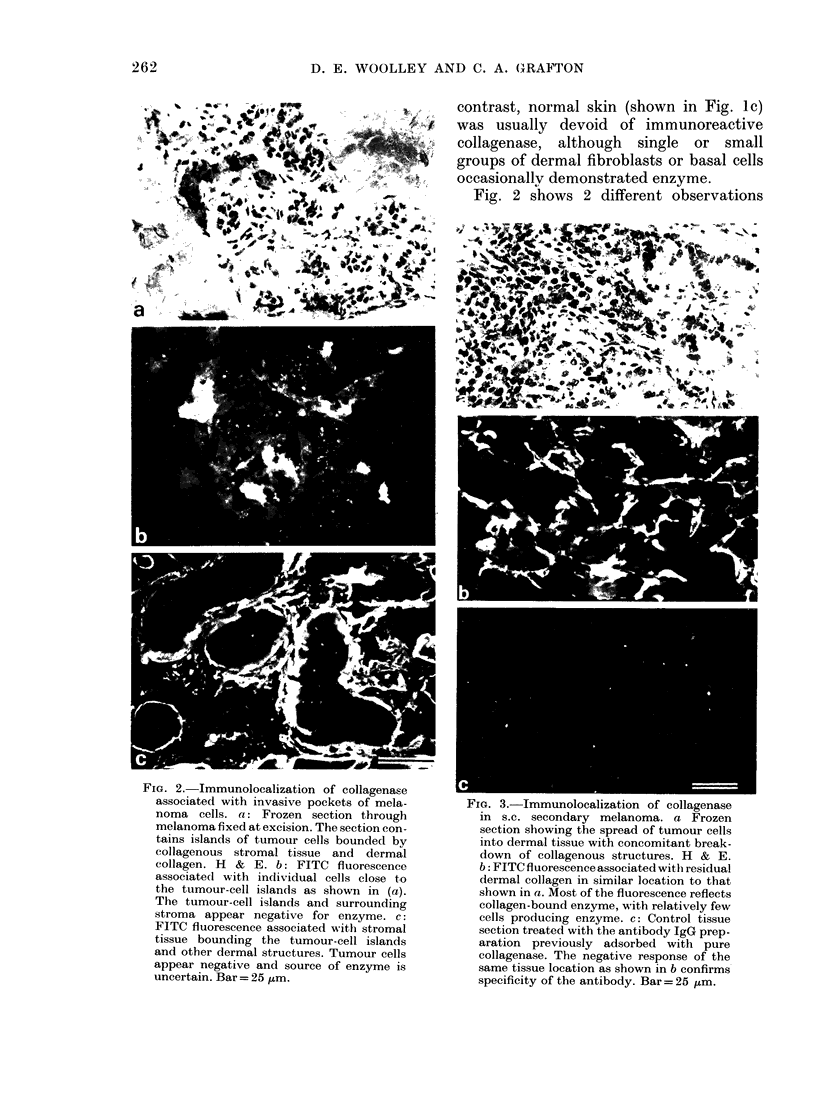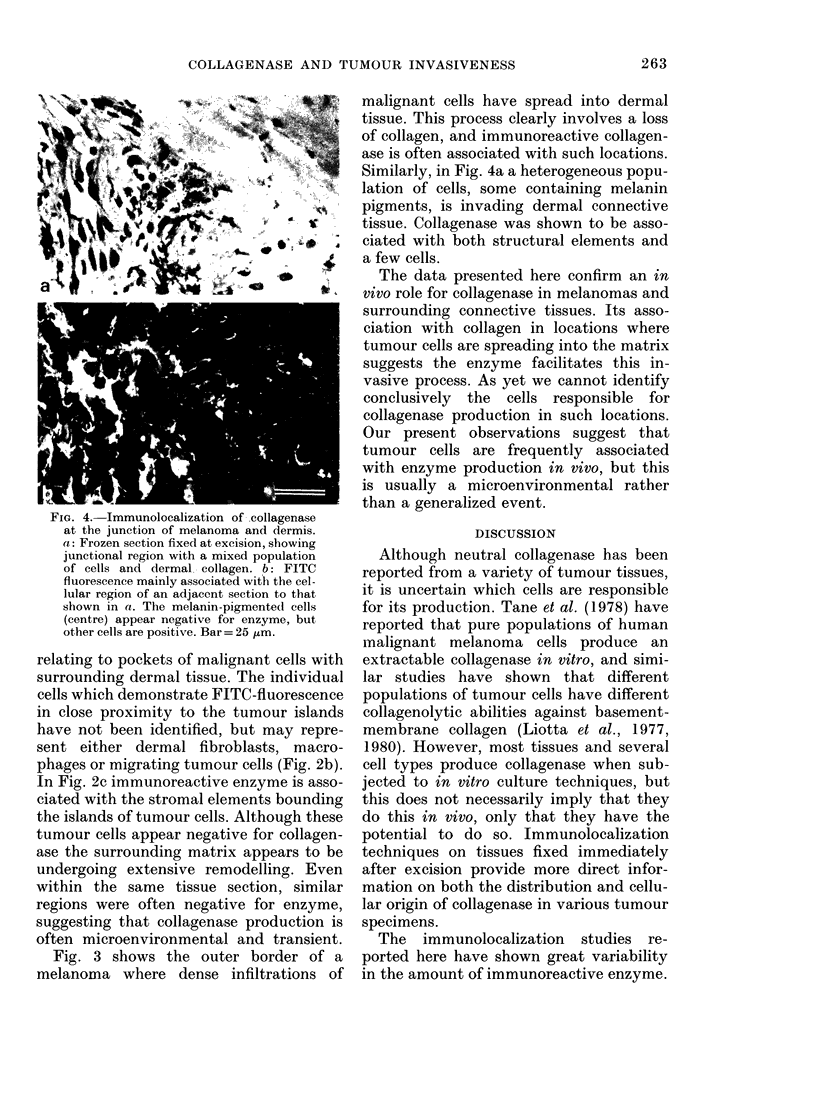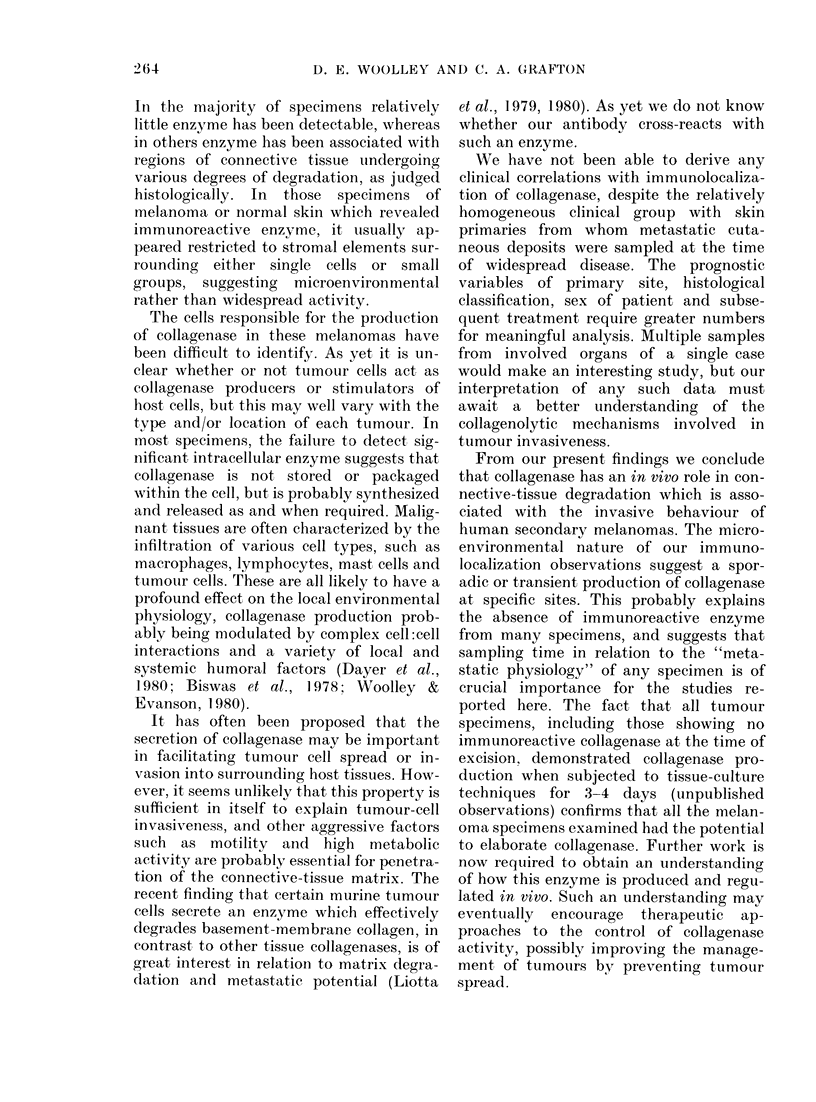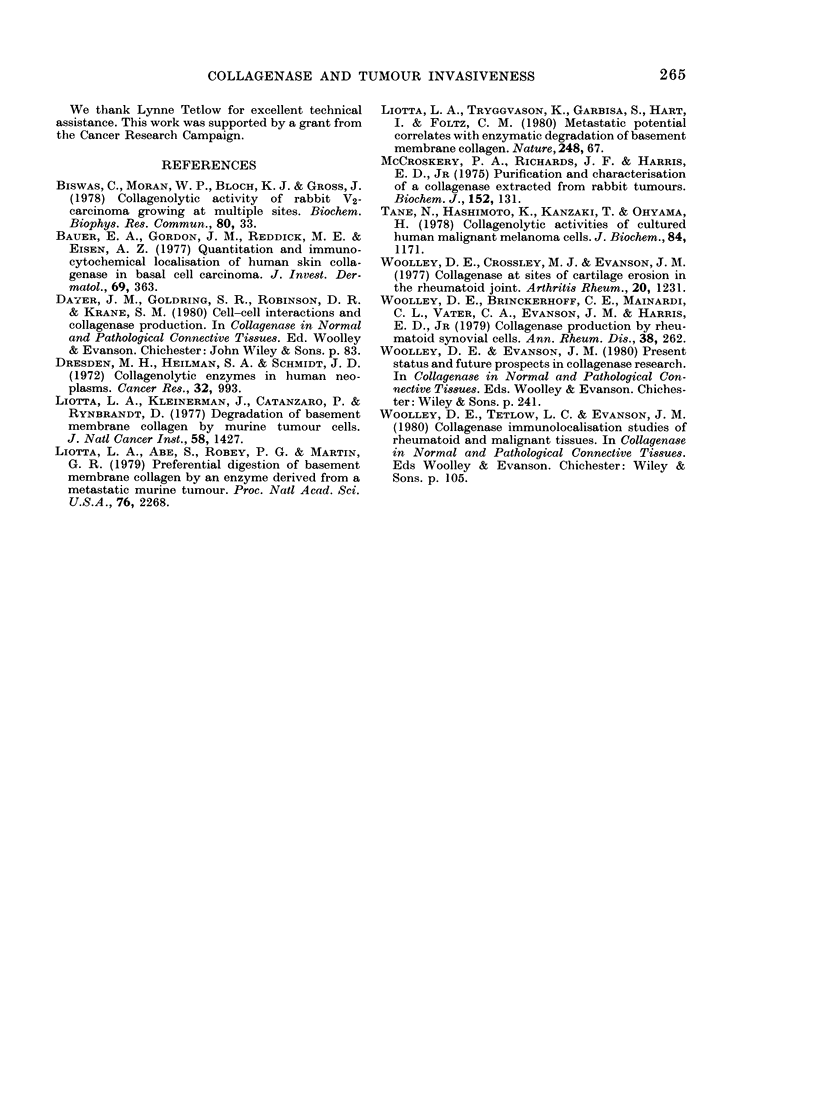# Collagenase immunolocalization studies of cutaneous secondary melanomas.

**DOI:** 10.1038/bjc.1980.225

**Published:** 1980-08

**Authors:** D. E. Woolley, C. A. Grafton

## Abstract

**Images:**


Br. J. Cancer (1980) 42, 260

### COLLAGENASE IMMUNOLOCALIZATION STUDIES OF CUTANEOUS SECONDARY MELANOMAS

D. E. WOOLLEY* AND C. A. GRAFTON†

*From the *University Department of Medicine, University Hospital of South Manchester, and the †C.R.C. Department of Medical Oncology, Christie Hospital, Manchester M20*

Received 31 March 1980 Accepted 9 May 1980

**Summary.—Immunoreactive collagenase has been demonstrated in 5/14 specimens of cutaneous secondary melanomas. In contrast, very little enzyme was seen in 10 specimens of normal human skin. All specimens were fixed within minutes of excision. These findings support the hypothesis that collagenase facilitates connective-tissue breakdown which is associated with tumour invasiveness and metastatic spread.**

THE PRECISE FACTORS which enable tumour cells to detach from each other, and to penetrate local physical barriers presented by the extracellular matrix, remain obscure. Several groups have proposed that some tumours and malignant cells secrete matrix-degrading or collagenolytic enzymes to facilitate their invasion of surrounding tissues (for review see Woolley *et al.*, 1980). This has been supported by reports of collagenolytic activity obtained either from tissue culture or the extraction of various tumours, and such studies have suggested the involvement of neutral collagenase in tumour invasion (*e.g.* Dresden *et al.*, 1972; McCroskery *et al.*, 1975; Bauer *et al.*, 1977; Liotta *et al.*, 1979). However, the cellular origin of collagenase has been in doubt because of the heterogeneity of most tumour specimens. One approach to this problem is the use of immunolocalization techniques using an antibody specific to human collagenase. We have therefore examined specimens of cutaneous melanomas and normal skin for immunoreactive collagenase, and have demonstrated that the enzyme is more frequently found in malignant tissues, especially in locations which histologically show signs of collagen loss.

### MATERIALS AND METHODS

*Collagenase antibody.*—A specific antibody to human collagenase has been raised in sheep and characterized by double diffusion, enzyme inhibition and immunoelectrophoretic techniques (Woolley *et al.*, 1977, 1979, 1980). Collagenases obtained from various specimens of cultured gastric adenocarcinomas or skin melanomas showed cross-reactivity with the antibody, as demonstrated by double diffusion (Woolley *et al.*, 1980). Previous studies have shown that the antibody has poor reactivity with latent enzyme or collagenase inhibited by its natural inhibitors, and we believe that most of our immunofluorescence observations reflect active, often collagen-bound enzyme (Woolley *et al.*, 1979, 1980).

*Immunolocalization.*—The specific sheep IgG antibody was separated from other immunoglobulins by affinity chromatography on a collagenase–Sepharose 4B column and eluted with 0·05M sodium citrate buffer (pH 3·2). The purified immune IgG was dialysed against phosphate-buffered saline (PBS) containing 0·02% sodium azide as preservative and used at a concentration of 50–100 $\mu$g IgG/ml.

Operative specimens of cutaneous melan-

Address for correspondence: Dr D. E. Woolley, Department of Medicine, University Hospital of South Manchester, Nell Lane, West Didsbury, Manchester M20 8LR.

oma were frozen in liquid $N_2$ within a few minutes of excision and stored at $-70°C$. Frozen sections (6–7 $\mu m$) were fixed for 1 min in freshly prepared 4% formalin, washed in PBS and examined by the indirect method of immunofluorescence, using the collagenase antibody in conjunction with FITC-labelled rabbit (anti-sheep IgG) immunoglobulins as described previously (Woolley *et al.*, 1977). Control tissue sections exposed to (a) non-immune sheep IgG in place of antibody, (b) immune sheep IgG previously adsorbed with pure collagenase, and (c) the FITC-conjugated antibody alone, accompanied each fluorescence study and were consistently negative.

Tissue sections were examined by incident fluorescence microscopy, using a Vickers M41 Photoplan microscope fitted with two FITC No. 5 interference exciter filters and a 200W mercury-vapour lamp. Fluorescence micrographs were made with Kodak Ektachrome ASA 200 film. Frozen sections from all tissue specimens were also stained with haematoxylin and eosin or van Gieson's/celestine blue for light microscopy.

*Melanoma specimens.*—All melanoma specimens were obtained from patients with skin primaries treated by wide excision with or without grafting, and block dissection. The disease biopsy specimens were obtained after a variable interval from primary treatment, and always in the presence of disseminated disease. One specimen was a local recurrence, the remainder were metastases: 2 from regional nodes and 11 from skin nodules.

The specimens of control skin were obtained from patients undergoing routine surgical procedures. The site of the skin sample varied according to the procedure which included mammaplasty, amputation and plastic surgery.

### RESULTS

Fourteen specimens of human cutaneous melanoma were subjected to collagenase immunolocalization, and positive findings were observed in 5. In contrast, very little immunoreactive enzyme was found in 10 specimens of normal skin. All specimens were fixed within minutes of surgery.

Fig. 1 shows 2 examples of skin containing infiltrations of tumour cells where immunoreactive collagenase is associated with the dermal tissue. The regions shown were in close association with invading secondary melanoma. Most of the fluorescence is associated with the dermal collagen and stromal elements bounding the islands of tumour cells. There is no significant staining of tumour cells or the epithelium. In

FIG. 1.—Immunolocalization of collagenase in s.c. secondary melanomas and normal skin. *a* and *b*: FITC fluorescence associated with dermal collagen and the stromal structures bounding the pockets of melanoma (Me). Deeper regions of the dermis (Der) and the epithelium (Ep) are negative for enzyme, as are most of the tumour-cell islands. *c*: Frozen section of normal skin showing no evidence of immunoreactive collagenase. Bar = 50 $\mu m$.

contrast, normal skin (shown in Fig. 1c) was usually devoid of immunoreactive collagenase, although single or small groups of dermal fibroblasts or basal cells occasionally demonstrated enzyme.

Fig. 2 shows 2 different observations

FIG. 2.—Immunolocalization of collagenase associated with invasive pockets of melanoma cells. *a*: Frozen section through melanoma fixed at excision. The section contains islands of tumour cells bounded by collagenous stromal tissue and dermal collagen. H & E. *b*: FITC fluorescence associated with individual cells close to the tumour-cell islands as shown in (*a*). The tumour-cell islands and surrounding stroma appear negative for enzyme. *c*: FITC fluorescence associated with stromal tissue bounding the tumour-cell islands and other dermal structures. Tumour cells appear negative and source of enzyme is uncertain. Bar = 25 $\mu$m.

FIG. 3.—Immunolocalization of collagenase in s.c. secondary melanoma. *a* Frozen section showing the spread of tumour cells into dermal tissue with concomitant breakdown of collagenous structures. H & E. *b*: FITC fluorescence associated with residual dermal collagen in similar location to that shown in *a*. Most of the fluorescence reflects collagen-bound enzyme, with relatively few cells producing enzyme. *c*: Control tissue section treated with the antibody IgG preparation previously adsorbed with pure collagenase. The negative response of the same tissue location as shown in *b* confirms specificity of the antibody. Bar = 25 $\mu$m.

FIG. 4.—Immunolocalization of collagenase at the junction of melanoma and dermis. *a*: Frozen section fixed at excision, showing junctional region with a mixed population of cells and dermal collagen. *b*: FITC fluorescence mainly associated with the cellular region of an adjacent section to that shown in *a*. The melanin-pigmented cells (centre) appear negative for enzyme, but other cells are positive. Bar = 25 μm.

relating to pockets of malignant cells with surrounding dermal tissue. The individual cells which demonstrate FITC-fluorescence in close proximity to the tumour islands have not been identified, but may represent either dermal fibroblasts, macrophages or migrating tumour cells (Fig. 2b). In Fig. 2c immunoreactive enzyme is associated with the stromal elements bounding the islands of tumour cells. Although these tumour cells appear negative for collagenase the surrounding matrix appears to be undergoing extensive remodelling. Even within the same tissue section, similar regions were often negative for enzyme, suggesting that collagenase production is often microenvironmental and transient.

Fig. 3 shows the outer border of a melanoma where dense infiltrations of malignant cells have spread into dermal tissue. This process clearly involves a loss of collagen, and immunoreactive collagenase is often associated with such locations. Similarly, in Fig. 4a a heterogeneous population of cells, some containing melanin pigments, is invading dermal connective tissue. Collagenase was shown to be associated with both structural elements and a few cells.

The data presented here confirm an *in vivo* role for collagenase in melanomas and surrounding connective tissues. Its association with collagen in locations where tumour cells are spreading into the matrix suggests the enzyme facilitates this invasive process. As yet we cannot identify conclusively the cells responsible for collagenase production in such locations. Our present observations suggest that tumour cells are frequently associated with enzyme production *in vivo*, but this is usually a microenvironmental rather than a generalized event.

### DISCUSSION

Although neutral collagenase has been reported from a variety of tumour tissues, it is uncertain which cells are responsible for its production. Tane *et al.* (1978) have reported that pure populations of human malignant melanoma cells produce an extractable collagenase *in vitro*, and similar studies have shown that different populations of tumour cells have different collagenolytic abilities against basement-membrane collagen (Liotta *et al.*, 1977, 1980). However, most tissues and several cell types produce collagenase when subjected to *in vitro* culture techniques, but this does not necessarily imply that they do this *in vivo*, only that they have the potential to do so. Immunolocalization techniques on tissues fixed immediately after excision provide more direct information on both the distribution and cellular origin of collagenase in various tumour specimens.

The immunolocalization studies reported here have shown great variability in the amount of immunoreactive enzyme.

In the majority of specimens relatively little enzyme has been detectable, whereas in others enzyme has been associated with regions of connective tissue undergoing various degrees of degradation, as judged histologically. In those specimens of melanoma or normal skin which revealed immunoreactive enzyme, it usually appeared restricted to stromal elements surrounding either single cells or small groups, suggesting microenvironmental rather than widespread activity.

The cells responsible for the production of collagenase in these melanomas have been difficult to identify. As yet it is unclear whether or not tumour cells act as collagenase producers or stimulators of host cells, but this may well vary with the type and/or location of each tumour. In most specimens, the failure to detect significant intracellular enzyme suggests that collagenase is not stored or packaged within the cell, but is probably synthesized and released as and when required. Malignant tissues are often characterized by the infiltration of various cell types, such as macrophages, lymphocytes, mast cells and tumour cells. These are all likely to have a profound effect on the local environmental physiology, collagenase production probably being modulated by complex cell:cell interactions and a variety of local and systemic humoral factors (Dayer *et al.*, 1980; Biswas *et al.*, 1978; Woolley & Evanson, 1980).

It has often been proposed that the secretion of collagenase may be important in facilitating tumour cell spread or invasion into surrounding host tissues. However, it seems unlikely that this property is sufficient in itself to explain tumour-cell invasiveness, and other aggressive factors such as motility and high metabolic activity are probably essential for penetration of the connective-tissue matrix. The recent finding that certain murine tumour cells secrete an enzyme which effectively degrades basement-membrane collagen, in contrast to other tissue collagenases, is of great interest in relation to matrix degradation and metastatic potential (Liotta *et al.*, 1979, 1980). As yet we do not know whether our antibody cross-reacts with such an enzyme.

We have not been able to derive any clinical correlations with immunolocalization of collagenase, despite the relatively homogeneous clinical group with skin primaries from whom metastatic cutaneous deposits were sampled at the time of widespread disease. The prognostic variables of primary site, histological classification, sex of patient and subsequent treatment require greater numbers for meaningful analysis. Multiple samples from involved organs of a single case would make an interesting study, but our interpretation of any such data must await a better understanding of the collagenolytic mechanisms involved in tumour invasiveness.

From our present findings we conclude that collagenase has an *in vivo* role in connective-tissue degradation which is associated with the invasive behaviour of human secondary melanomas. The microenvironmental nature of our immunolocalization observations suggest a sporadic or transient production of collagenase at specific sites. This probably explains the absence of immunoreactive enzyme from many specimens, and suggests that sampling time in relation to the "metastatic physiology" of any specimen is of crucial importance for the studies reported here. The fact that all tumour specimens, including those showing no immunoreactive collagenase at the time of excision, demonstrated collagenase production when subjected to tissue-culture techniques for 3–4 days (unpublished observations) confirms that all the melanoma specimens examined had the potential to elaborate collagenase. Further work is now required to obtain an understanding of how this enzyme is produced and regulated *in vivo*. Such an understanding may eventually encourage therapeutic approaches to the control of collagenase activity, possibly improving the management of tumours by preventing tumour spread.

We thank Lynne Tetlow for excellent technical assistance. This work was supported by a grant from the Cancer Research Campaign.

### REFERENCES

BISWAS, C., MORAN, W. P., BLOCH, K. J. & GROSS, J. (1978) Collagenolytic activity of rabbit $V_2$-carcinoma growing at multiple sites. *Biochem. Biophys. Res. Commun.*, **80**, 33.

BAUER, E. A., GORDON, J. M., REDDICK, M. E. & EISEN, A. Z. (1977) Quantitation and immunocytochemical localisation of human skin collagenase in basal cell carcinoma. *J. Invest. Dermatol.*, **69**, 363.

DAYER, J. M., GOLDRING, S. R., ROBINSON, D. R. & KRANE, S. M. (1980) Cell–cell interactions and collagenase production. In *Collagenase in Normal and Pathological Connective Tissues*. Ed. Woolley & Evanson. Chichester: John Wiley & Sons. p. 83.

DRESDEN, M. H., HEILMAN, S. A. & SCHMIDT, J. D. (1972) Collagenolytic enzymes in human neoplasms. *Cancer Res.*, **32**, 993.

LIOTTA, L. A., KLEINERMAN, J., CATANZARO, P. & RYNBRANDT, D. (1977) Degradation of basement membrane collagen by murine tumour cells. *J. Natl Cancer Inst.*, **58**, 1427.

LIOTTA, L. A., ABE, S., ROBEY, P. G. & MARTIN, G. R. (1979) Preferential digestion of basement membrane collagen by an enzyme derived from a metastatic murine tumour. *Proc. Natl Acad. Sci. U.S.A.*, **76**, 2268.

LIOTTA, L. A., TRYGGVASON, K., GARBISA, S., HART, I. & FOLTZ, C. M. (1980) Metastatic potential correlates with enzymatic degradation of basement membrane collagen. *Nature*, **248**, 67.

MCCROSKERY, P. A., RICHARDS, J. F. & HARRIS, E. D., JR (1975) Purification and characterisation of a collagenase extracted from rabbit tumours. *Biochem. J.*, **152**, 131.

TANE, N., HASHIMOTO, K., KANZAKI, T. & OHYAMA, H. (1978) Collagenolytic activities of cultured human malignant melanoma cells. *J. Biochem.*, **84**, 1171.

WOOLLEY, D. E., CROSSLEY, M. J. & EVANSON, J. M. (1977) Collagenase at sites of cartilage erosion in the rheumatoid joint. *Arthritis Rheum.*, **20**, 1231.

WOOLLEY, D. E., BRINCKERHOFF, C. E., MAINARDI, C. L., VATER, C. A., EVANSON, J. M. & HARRIS, E. D., JR (1979) Collagenase production by rheumatoid synovial cells. *Ann. Rheum. Dis.*, **38**, 262.

WOOLLEY, D. E. & EVANSON, J. M. (1980) Present status and future prospects in collagenase research. In *Collagenase in Normal and Pathological Connective Tissues*. Eds. Woolley & Evanson. Chichester: Wiley & Sons. p. 241.

WOOLLEY, D. E., TETLOW, L. C. & EVANSON, J. M. (1980) Collagenase immunolocalisation studies of rheumatoid and malignant tissues. In *Collagenase in Normal and Pathological Connective Tissues*. Eds Woolley & Evanson. Chichester: Wiley & Sons. p. 105.